# Disclosing the temperature of columnar jointing in lavas

**DOI:** 10.1038/s41467-018-03842-4

**Published:** 2018-04-12

**Authors:** Anthony Lamur, Yan Lavallée, Fiona E. Iddon, Adrian J. Hornby, Jackie E. Kendrick, Felix W. von Aulock, Fabian B. Wadsworth

**Affiliations:** 1 Experimental Volcanology Laboratory, Department of Earth, Ocean and Ecological Sciences, University of Liverpool, 4 Brownlow Street, Liverpool, L69 3GP UK; 2 Department of Earth Sciences, University of Cambridge, Downing Street, Cambridge, CB2 3EQ UK; 3 Present Address: Department of Earth Sciences, Durham University, Science Labs, Elvet Hill, DH1 3LE Durham, UK

## Abstract

Columnar joints form by cracking during cooling-induced contraction of lava, allowing hydrothermal fluid circulation. A lack of direct observations of their formation has led to ambiguity about the temperature window of jointing and its impact on fluid flow. Here we develop a novel thermo-mechanical experiment to disclose the temperature of columnar jointing in lavas. Using basalts from Eyjafjallajökull volcano (Iceland) we show that contraction during cooling induces stress build-up below the solidus temperature (980 °C), resulting in localised macroscopic failure between 890 and 840 °C. This temperature window for incipient columnar jointing is supported by modelling informed by mechanical testing and thermal expansivity measurements. We demonstrate that columnar jointing takes place well within the solid state of volcanic rocks, and is followed by a nonlinear increase in system permeability of <9 orders of magnitude during cooling. Columnar jointing may promote advective cooling in magmatic-hydrothermal environments and fluid loss during geothermal drilling and thermal stimulation.

## Abstract (web summary)

Columnar joints in lavas form during cooling, but the temperature this occurs at is unclear. Here, the authors perform thermo-mechanical experiments on basaltic rocks to examine the temperature of columnar joints in lavas and find that failure occurs at 890–840 °C, which is below the solidus temperature of 980 °C.

## Introduction

Columnar joints form by cracking due to cooling-driven contraction of igneous rocks^1^, which results in tensile failure^2^. Their presence in the rock record has long represented one of the most awe-inspiring geological features^3^ and their regular geometry has challenged our understanding of pattern ordering during thermal contraction^4^. To date, the temperature of their formation has remained unconstrained, although it holds thermo-mechanical information key to resolving the cooling history of volcanic rocks^5,6^ and intrusive magma bodies^7^. Columnar joints are permeable structures that play an important role in fluid circulation in the crust, exerting controls on heat transfer^7,8^, resource transport and ore deposition^9^, geothermal and hydrothermal reservoirs^10^ as well as rock alteration, and degradation of rock mechanical properties^11^.

**Fig. 1:**
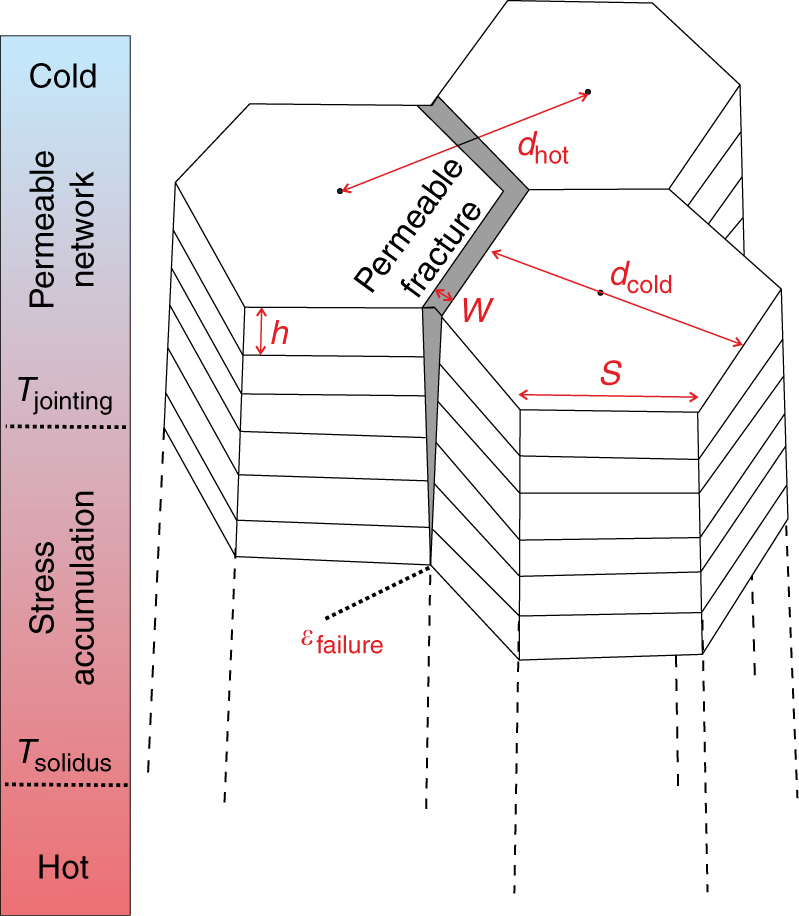
Columnar jointing model. The sketch shows that tensile fractures ensue from strain accumulation, induced by thermal contraction of a length between two focal points (*d*_hot_), exceeding the tensile strain limit of the rock (*ε*_failure_). The length difference between *d*_hot_ and the columnar joint diameter (*d*_cold_) is the width of the permeable fracture (grey area, *w*) along length *s* available for fluid infiltration

Columnar joints develop in cooling intrusive and extrusive volcanic rocks, irrespective of their chemical composition or emplacement environments, and have recently been discovered on Mars^12^. Structurally, columnar-jointed rocks classically exhibit two jointing facies: in an idealised system, a cooling unit is characterised by a lower colonnade with linear and parallel columns; and an overlying entablature with curved and irregular columns, which may be superimposed by an upper colonnade^13,14^. In complex bodies stress distribution can disorder column formation^5,14^ and occasional absence of the upper colonnade has been attributed to erosion or intense cooling regimes accentuated by abundant water incursion in a flow’s interior^15^. Colonnades exhibit quasi-hexagonal fracture patterns, bounded by striae (Fig. 1), the spacing of which has been shown to scale with column width, likely reflecting the cooling history of the flow^16,17^. The quasi-hexagonal fracture geometry has been ascribed to thermal contraction induced by conductive cooling, occasionally enhanced by water infiltration of the fracture network^2,16,18–20^. The hypothesis remains that upon cooling, tensile stress accumulates elastically^21^, generating a random network of micro-fractures, which slowly develops into a more ordered polygonal set of mode-I tensile macro-fractures^4^ (Fig. 1). The formation of a permeable fracture network then increases the infiltration and transport potential of fluids in the cooling body, contributing to the development of entablature^22^. It has previously been proposed that stress build-up and fracturing takes place during rapid cooling beyond the glass transition of the melt inside crystallising lavas (that is, at super-solidus temperatures where lava is still partially molten)^5,22–24^. Semi-circular petrographic structures, present in some columnar joints, are interpreted to result from melt segregation driven by contraction and fracturing triggered during crystallisation^1,25^. Cross cutting of the semi-circular structure by the fracture has been used to contest fracturing of melt (undergoing the glass transition); instead melt segregation has been demonstrated to result from changes in the physical and rheological properties of melts during crystallisation^6^. The quenching of melt to glass relies on a relatively rapid cooling rate and predominantly high viscosities, which prevent complete crystallisation^26^; thus, the glass transition is more likely to be met in silicic lavas than in mafic lavas. In basaltic lava flows, vitrification is restricted to areas with very rapid cooling: the upper millimetres of a flow emplaced in air^27^, the fracture surface of entablatures, and in hyaloclastite and pillow lavas erupted in sub-aqueous or sub-glacial environments^22,28,29^. Here we propose that columnar joints form in the purely elastic regime of rocks; and that further cooling beyond their incipient formation results in fracture opening and therefore constructs and extends the permeable network that channels fluid flow and accentuates heat exchange.

## Results

### Combined thermo-mechanical jointing experiments

**Fig. 2:**
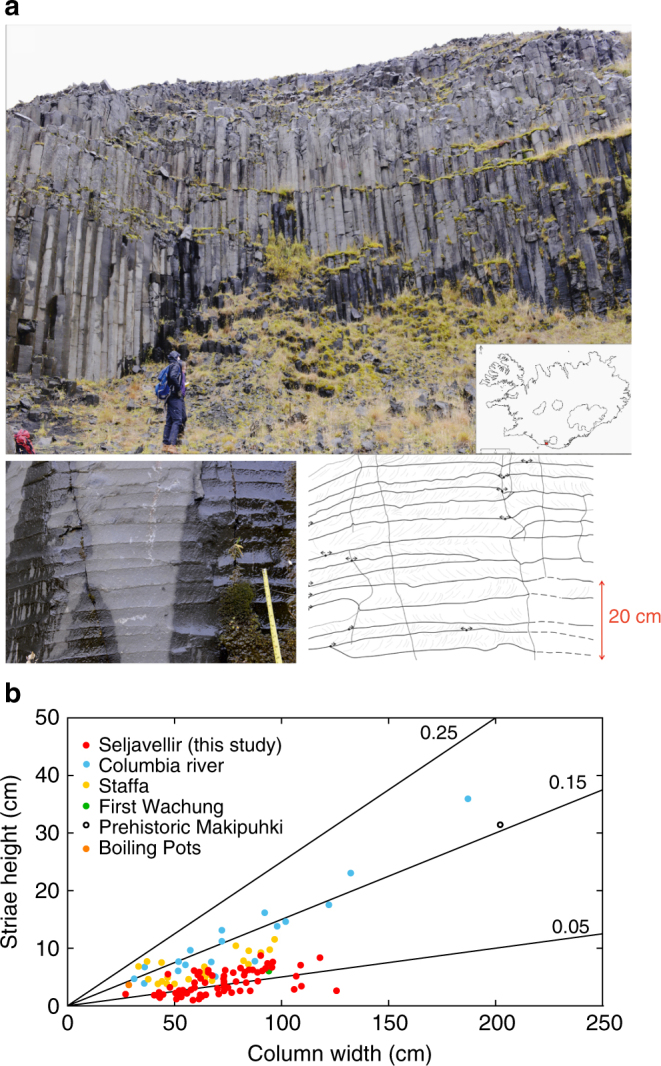
Columnar-jointed basalt at Seljavellir. **a** Columnar joint outcrop locality complemented by a close-up photo and sketch of striae along a colonnade. The exposure is characterised by quadratic to heptagonal cross-sectional patterns. The fracture surfaces reveal striae, exhibiting both a rough and a smooth portion. **b** Geometrical relationship between the height of Striae (*h*) and the column width (*d*_cold_) as shown schematically in Fig. 1. The data are plotted against other columnar-jointed lavas from Columbia River^16,17^, Staffa^5^, First Wachung, Prehistoric Mahipuhki and Boiling Pots^24^

**Fig. 3:**
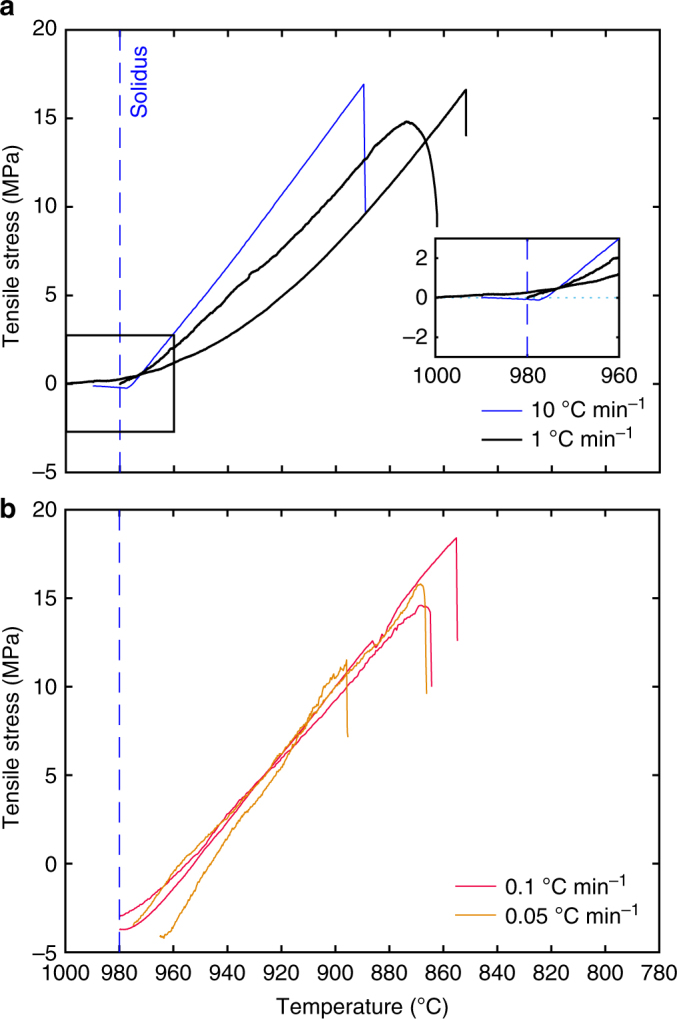
Mechanics of columnar jointing. Tensile stress builds up as a rock, locked into a fixed length, cools at a set rate of 0.05 (orange), 0.1 (red), 1 (black) and 10 °C min^−1^ (blue), starting with **a** no applied normal stress and **b** 5 MPa normal stress. The dashed blue line denotes the solidus temperature (980 °C)

To test the thermo-mechanics of columnar joints we developed a novel experimental setup that allows us to directly observe fracturing in cooling lavas (Supplementary Fig. 1). In the experiment, a tensile fracture is induced by cooling a cylindrical sample of rock from its solidus temperature while locking the ends of the cylinder in position, initially imposing 0 or 5 MPa of uniaxial compressive stress, perpendicular to the experimentally generated fracture plane (thus simulating normal stress at depth). This setup is designed to simulate elastic stress accumulation by thermal contraction between the static centres of two colonnades. Experiments were conducted on a typical, micro-crystalline basaltic lava from a jointed body at Seljavellir, at the base of Eyjafjallajökull, Iceland (Fig. 2a). The colonnades exhibit quasi-hexagonal fracture patterns, jointed into columns ranging between 30 and 130 cm across. The approximately regular spacing of striae, which sometimes pinch in and out laterally (Fig. 2a), scales with the column width^16,17,24^ (Fig. 2b). The basalt consists of plagioclase, olivine, occasional pyroxenes and iron oxide crystals (Supplementary Figs. 2 and 3), set in a micro-crystalline groundmass of the same mineralogy, which hosts no interstitial glass (Supplementary Fig. 3). The basalt has a solidus temperature of 980 °C (at 1 bar and fO_2_ between nickel-nickel oxide (NNO) and quartz-fayalite-magnetite (QFM) oxygen buffers) estimated by MELTS^30^ on the basis of the bulk rock chemistry (Supplementary Table 1); this was supported visually by the onset of melting of iron oxides at the starting temperature of the experiments. In the columnar jointing experiments, cooling resulted in tensile stress build-up from the solidus temperature down to temperatures of 890–840 °C, regardless of the imposed cooling rate and initial stress (Fig. 3). In this temperature range, the tensile stress accumulated (12–18 MPa) induced failure, resulting in the creation of a through-going fracture and accompanying stress drop. With further cooling, the fracture widened.

### Dilatometric measurements and mechanical testing

**Fig. 4:**
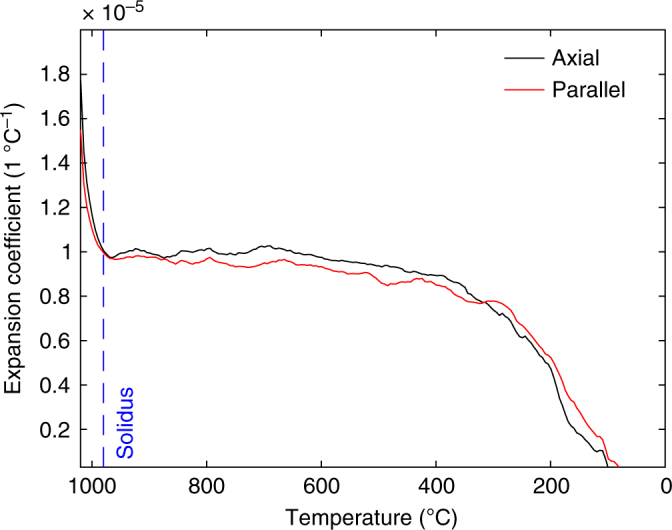
Thermal expansion coefficient. Dilatometric measurements showing the linear expansion coefficient of Seljavellir basalt, cored axially and parallel to the column, during cooling from 1020 °C. The dashed blue line denotes the solidus temperature (980 °C)

**Fig. 5:**
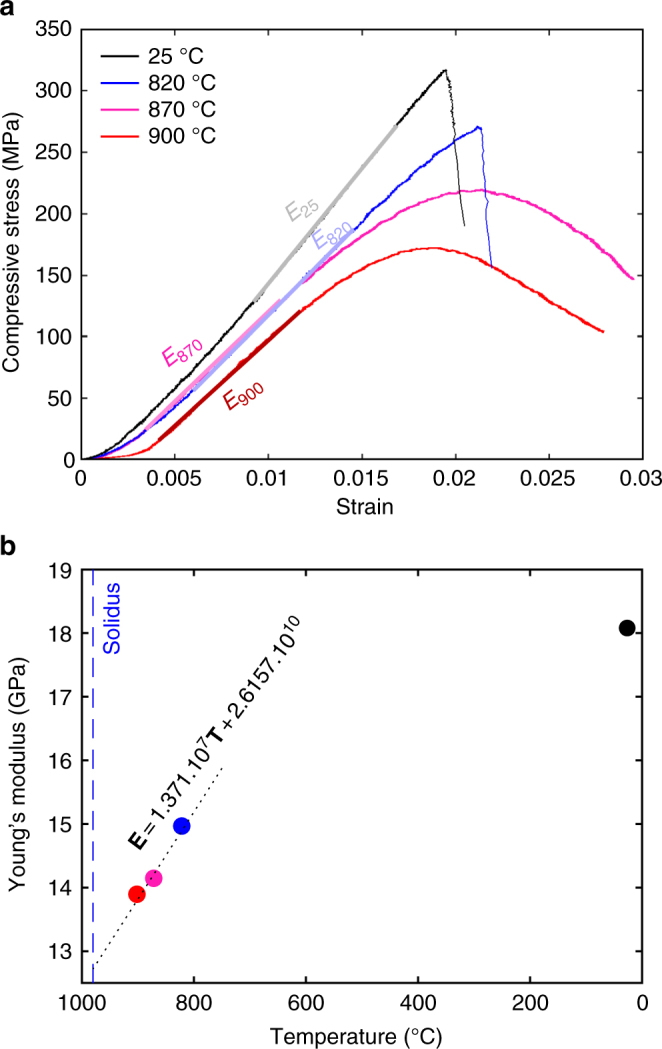
Young’s modulus variation with temperature. **a** Mechanical data obtained through uniaxial compressive testing at ambient temperature (black), 820 (blue), 870 (magenta) and 900 °C (red). Young’s moduli at the different temperatures (*E*_25_, *E*_820_, *E*_870_ and *E*_900_) are calculated using the linear (elastic) portion of each curve. **b** Young’s modulus values at each temperature, showing a linear evolution at high temperatures, as depicted by the dotted black line. The dashed blue line denotes the solidus temperature (980 °C)

**Fig. 6:**
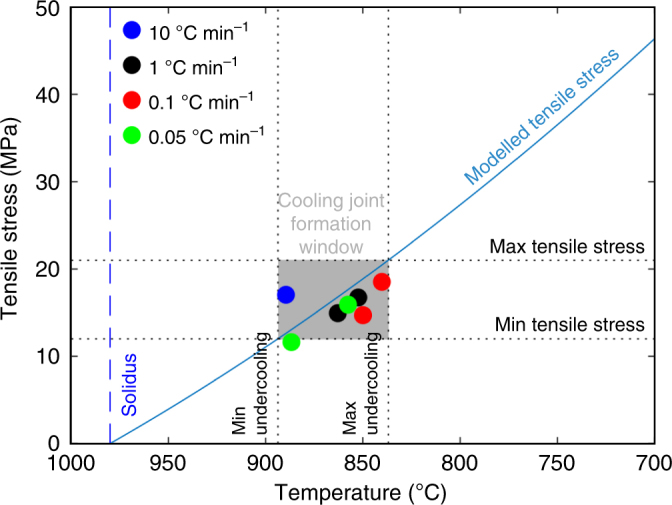
Predicted columnar jointing temperature window. The horizontal dotted black lines show the minimum and maximum strength limits obtained through Brazilian tensile tests. The solid blue line shows the calculated tensile stress build-up upon cooling. The intercepts (vertical dotted lines) between the calculated stress curve and the measured strength lines denote the cooling window necessary to achieve failure. The coloured dots represent the fracturing temperatures achieved during columnar jointing experiments. The dashed blue line denotes the solidus temperature (980 °C)

To ensure that this temperature window of columnar jointing is realistic, we support our analysis with a dilatometric and mechanical study to assess whether the dynamics of columnar jointing can be explained by comparison of two distinct, static tests. Dilatometric measurements revealed that the expansion coefficient, *α*, of the basalt tested is isotropic and linear in the temperature range of interest (400–980 °C), equating to ~10^−5^ °C^−1^, with rapid volumetric expansion at temperatures above 980 °C, which indicates melting and matches the solidus temperature estimated by MELTS^30^ (Fig. 4). Additionally, ambient and high-temperature (820–900 °C) uniaxial compressive strength tests conducted at a strain rate of 10^−5^ s^−1^ were used to define the temperature-dependence of the Young’s modulus **E** (Fig. 5a). **E** was found to evolve according to an empirical relationship ${\mathbf{E}} = p{\mathbf{T}} + E_0$ (where **E** is in Pa and **T** is in °C). Here *p* = 1.371 × 10^7^ °C^−1^ and *E*_0_ = 2.6157 × 10^10^ Pa in the high-temperature window of columnar jointing (Fig. 5b). Finally, ambient and high-temperature (600–940 °C) Brazilian tensile tests constrained the tensile strength of our samples to 12–21 MPa (Supplementary Fig. 4), in good agreement with the failure stress of 12–18 MPa recorded in the combined thermo-mechanical jointing experiments. Together, these thermo-mechanical constraints allow us to model the tensile stress build-up over a range of undercooling Δ*T*, via ${\mathbf{\sigma }}_{\mathbf{T}} = {\mathbf{E}}\alpha \Delta T$^31^. Our calculations suggest that the temperature of macroscopic failure, *T*_f_, is 87–144 °C below the solidus; that is, between 893 and 836 °C (Fig. 6)—a temperature window in excellent agreement with the results of columnar jointing experiments developed here (890–840 °C; Fig. 3).

### Modelling of fracture widening and fluid flow

Studies assessing the thermal history of magma reservoirs, sills and dykes often point towards arguably long cooling timescales if conduction alone is considered, and thus commonly infer the need for external fluid infiltration to increase the cooling efficiency of the magma body^2,7,8^. The thermo-mechanical constraints introduced here suggest that fluid infiltration may contribute to the thermal budget following columnar joint formation. Here the data reveal that after their formation at 890–840 °C, fractures would open by continued contraction proportional to an expansion coefficient of ~10^−5^ °C^−1^ down to 400 °C (and at a slower rate below this temperature; Fig. 3), thereby constructing the permeable network that allows fluid infiltration in an otherwise largely impermeable rock (measured at 5 × 10^−20^ m^2^ without fractures). Using the linear expansion coefficient *α* established for different temperatures (*T*) by dilatometric measurements, we model the evolution of fracture width, *w* due to contraction of the rock (between the centre of two columns with length *d*_hot_) when cooling below the fracturing temperature *T*_f_ (893–836 °C):

1 $$w\, = \,\alpha d_{\mathrm{hot}}\left[ {T_{\mathrm{f}}\, - \,T} \right]$$

**Fig. 7:**
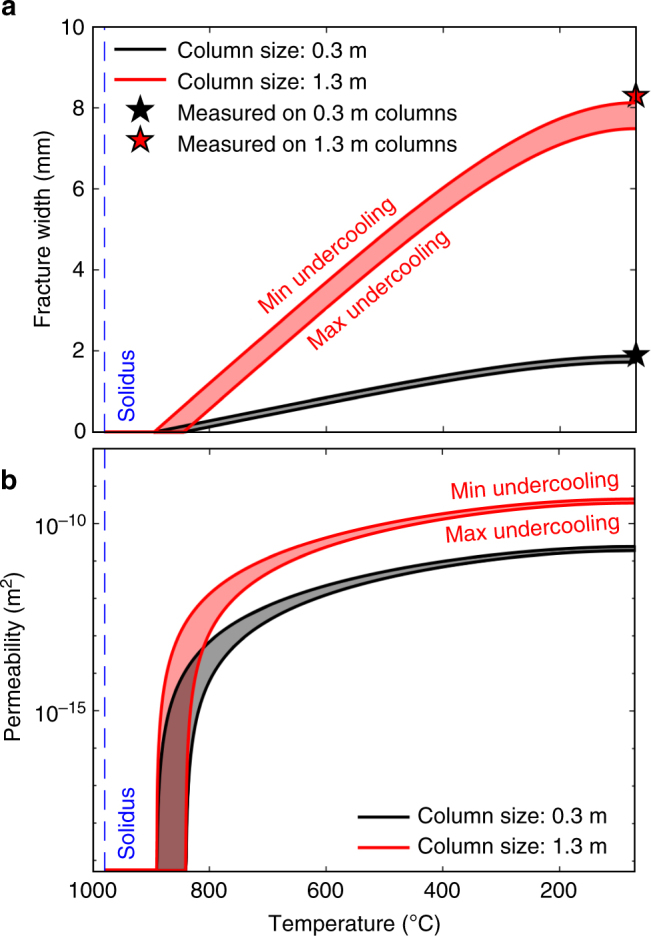
Fracture width and permeability evolution of columnar-jointed rocks. **a** Fracture width evolution in a magmatic body developing columns of 0.3 (black) and 1.3 m (red) diameter. The black and red stars show the average fracture width, measured in the field, for 0.3 and 1.3 m diameter columns, respectively. **b** Permeability increase due to induced fracture opening in a cooling system with 0.3 (black) and 1.3 m (red) diameter columns. In both **a** and **b** the dashed blue line denotes the solidus temperature (980 °C)

Here, we gauge the spectrum of *d*_hot_ using the range of column sizes observed at Seljavellir (*d*_cold_ = 0.3–1.3 m) as proxy. Our calculation of the final fracture width shows good agreement with values of 1.9 and 8.3 mm measured in the field (Fig. 7a). Knowing the fracture width at different temperatures allows us to predict the permeability of a fractured rock mass, *κ*_fr_ (in m^2^), contracting during cooling in the absence of stress, using a scaling for the permeability of fractured systems^32^. We modify this scaling to account for arrays of hexagonal columnar joints separated by fractures (see Methods) to find:

2 $$\kappa _{\mathrm{fr}}\, = \,\frac{{s\sqrt 3 \kappa _{\mathrm{h}}}}{{2w + s\sqrt 3 }}\, + \,\frac{{w^3}}{{12w + 6s\sqrt 3 }}$$

where *κ*_h_ is the permeability of the intact rock before jointing and *s* is the edge-length of the colonnade hexagons (in m). Thus, we calculate the permeability of columnar-jointed magmatic aureoles during cooling and show that the evolution is primarily, and highly dependent on the column size (Fig. 7b). Hence the formation of columnar joints may strongly influence hydrothermal circulation and therefore, the cooling history of magmatic bodies near the Earth’s surface and in fluid-rich environments characteristic of shallow volcanic, geothermal and hydrothermal systems.

## Discussion

Our results are consistent with incipient columnar joint formation at a relatively high temperature, yet within the range at which the magma body is fully elastic. Given that no stress accumulates at temperatures above the solidus (Fig. 3a, inset) and that an undercooling of at least 90 °C (from the solidus) is required to induce tensile fracturing in this basalt, we advance that columnar joints must form and propagate in the solid state, at high-to-moderate temperatures. This thermal constraint in the sub-solidus regime is in agreement with careful structural and petrographic observations recently put forth^6^, and thus suggests that the formation of columnar joints (at least those widespread in basaltic to basaltic-andesite lavas) by crossing of the glass transition of the melt^24^ can only happen in extremely rapidly cooled (more rapid than the rates herein), for example, in fluid-saturated environments.

Thermally induced columnar jointing leads to the construction of a fracture network with system permeability increasing non-linearly during cooling. The permeability gradient observed along a cooling column would allow sufficient water infiltration to trigger convection-driven cooling^8^ and eventually trigger quenching of liquid in the flow core, providing a mechanism for the formation of glass films observed along the entablatures^22^. Drastic permeability increase during cooling of jointing bodies (Fig. 7b) may also explain why the aureoles of shallow magma bodies serendipitously encountered by geothermal drilling^33,34^ have very steep temperature gradients over short distances of a few tens of metres^35,36^. Drilling of these magmatic aureoles has been characterised by strong loss in drilling fluid circulation^37^; the results here suggest that efficient cooling by injection of fluids at ~80 °C in a magmatic aureole at ca. 850 °C would have caused thermal jointing and a significant widening of fractures to allow drilling fluid circulation loss during drilling activity. Our results suggest that numerical simulations of natural cooling systems should incorporate thermo-mechanical constraints based on well-constrained experimental work to refine the limits of fluid circulation in volcanic, geothermal and hydrothermal environments.

## Methods

### Combined thermo-mechanical jointing experiments

Combined thermo-mechanical jointing experiments, mimicking columnar jointing, were conducted in the Experimental Volcanology and Geothermal Laboratory at the University of Liverpool in a 5969 Instron press equipped with a custom furnace designed by Severn Thermal Solutions, which permits infrared (IR) imaging of samples through a sapphire window. For this purpose, 170 mm-long samples, 16 mm in diameter were cored out and the central 35 mm length of the sample was ground to a thinner dog-bone geometry with a diameter of 8–10 mm, conferring a stress accuracy of ±0.01 MPa (using the standard error of estimate method, machine accuracy and sample geometry). During sample heating (at 5 °C min^−1^) the grips were allowed to freely retract with sample expansion. Once the temperature equilibrated (after 30 min), the grips were locked in position and the furnace was programmed to cool at set rates of 0.05, 0.1, 1 or 10 °C min^−1^, whilst the press monitored tensile stress incurred by sample contraction. A FLIR SC6540 IR thermographic camera imaged sample temperature in response to the set rate of the furnace during cooling.

### Dilatometry

Dilatometric measurements were performed at the University of Liverpool in a Thermo-Mechanical Analyser 402F1—Hyperion from Netzsch GmbH. The expansion and contraction of a 6 mm diameter by 5 mm-high cylindrical sample was monitored at a heating and cooling rate of 2 °C min^−1^ (up to 1020 °C) with a spatial resolution of 0.125 nm, whilst applying 3 mN load. The sample expansion was corrected for sample holder expansion by subtracting a baseline run on a sample of standard alumina at the same heating and cooling rate. The corrected values of length changes were used to calculate the linear expansion coefficient.

### Indirect tensile testing

Brazilian tests at room and high temperatures were conducted to assess the tensile strength^38^ in a 8800 Instron press equipped with a furnace designed by Severn Thermal Solutions, in the Experimental Volcanology and Geothermal Laboratory at the University of Liverpool. Disc-shaped samples with a diameter of 40 mm and a thickness of 20 mm were placed edge-on in the press and heated at 2 °C min^−1^ to the desired sample temperature (600–940 °C), conferring a stress accuracy of ±0.02 MPa (using the standard error of estimate method, machine accuracy and sample geometry). After 1 h of thermal equilibration at the target temperature, the cylindrical sample was diametrically compressed at a rate of 4 × 10^−4^ mm s^−1^ with force and deformation monitored until complete failure. Room temperature tests were conducted at the same rate.

### Young’s modulus measurements

The temperature-dependence of the static Young’s modulus was constrained by conducting a series of uniaxial compressive strength tests in a 8800 Instron press in the Experimental Volcanology and Geothermal Laboratory at the University of Liverpool. Cylindrical samples with a diameter of 10 mm and length of 20 mm were placed between the pistons of the press and heated at a rate of 2 °C min^−1^ to the desired sample temperatures (820–900 °C) centred around *T*_f_. Once thermally equilibrated (30 min), the cylinders were deformed at a rate of 10^−5^ s^−1^ and stress and strain were monitored. The linear portion of the stress–strain data was used to constrain the static Young’s modulus, with an accuracy of ±0.03 GPa (using the standard error of estimate method, machine accuracy, sample geometry and sample strength).

### Permeability determination

The permeability of the rock was measured in the Experimental Volcanology and Geothermal Laboratory at the University of Liverpool in a Sanchez hydrostatic vessel, using water pressures (in) of 2.1 MPa and (out) of 0.1 MPa (thus a pore pressure gradient, Δ*P* = 2 MPa), and 6 MPa confining pressure exerted by oil on a jacketed cylindrical sample (25 mm diameter). Water volumetric discharge rate, *Q* (in m^3^ s^−1^) was monitored and used to calculate the permeability, *κ*, via Darcy’s equation^39,40^:

3 $$\kappa \, = \,\frac{{Q\eta L}}{{A{\mathrm{\Delta }}P}}$$

where *A* is the sample cross-sectional area (m^2^), *L* the sample length (m) and *η* the viscosity of the fluid (0.001 Pa s for water).

### Modelling of fluid flow in a jointed body

To model the permeability, *κ*_s_, of a reservoir developing columnar joints, we use a simple scaling^32^ modified for a two-dimensional areal case:

4 $$\kappa _{\mathrm{s}}\, = \,\frac{{A_{\mathrm{h}}\kappa _{\mathrm{h}}}}{{A_{\mathrm{i}}}}\, + \,\frac{{A_{\mathrm{f}}\kappa _{\mathrm{f}}}}{{A_{\mathrm{i}}}}$$

where *κ*_h_ is the permeability of the intact rock, *A*_h_ is the two-dimensional top-area of impermeable columns perpendicular to the fracture propagation direction, *A*_f_ is the area of the fractures, *A*_i_ is the total area (as the sum of *A*_f_ and *A*_h_) and *κ*_f_ is the permeability of a fracture.

Considering the system shown in Fig. 1 and applying it to Seljavellir basalts, we define the following set of parameters. Assuming hexagonal columns, the area of a unit cell of two impermeable columns, *A*_h_, is given by:

5 $$A_{\mathrm{h}}\, = \,3\sqrt 3 s^2$$

The fractured area *A*_f_ is given by the six fracture edges in the unit cell of two hexagons as:

6 $$A_{\mathrm{f}}\, = \,6sw$$

Applying Poiseuille’s law for fluid flow in an infinite slot, we can derive the permeability of a fracture, *κ*_f_, as:

7 $$\kappa _{\mathrm{f}}\, = \,\frac{{w^2}}{{12}}$$

Substituting Eqs. 5–7 into Eq. 4, we find that the permeability of the jointing system follows a simple analytical form, which depends only on *κ*_h_, *s* and *w*, shown in Eq. 2.

### Data availability

The authors declare that all data supporting the findings of this study are available within the paper and its Supplementary Information files.

## Electronic supplementary material

Supplementary Information
