# Supplementary material for: Disclosing the temperature of columnar jointing in lavas
Source: Nat Commun. 2018 Apr 12;9:1432. doi: 10.1038/s41467-018-03842-4 (PMC5897544; doi:10.1038/s41467-018-03842-4)
Supplement: Supplementary file 1 — Supplementary Information [file 41467_2018_3842_MOESM1_ESM.pdf]

Supplementary Information

Disclosing the temperature of columnar jointing in lavas

Anthony Lamur, Yan Lavallée, Fiona E. Iddon, Adrian J. Hornby, Jackie E. Kendrick, Felix W. von Aulock & Fabian B. Wadsworth

Supplementary Figures

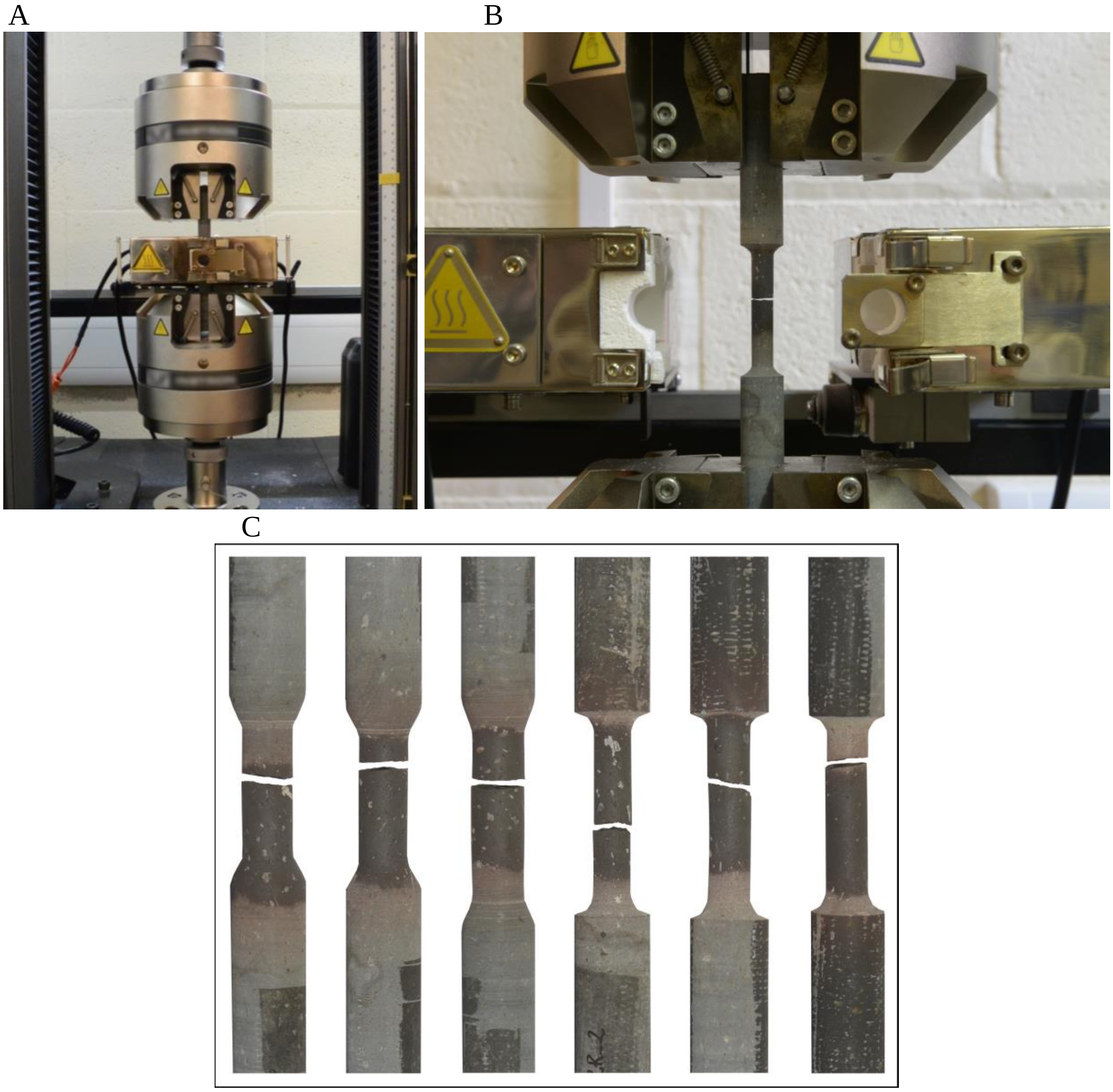

Supplementary Figure 1. Photograph of experimental setup and sample assembly. A) The sample is mounted between two grips held in an Instron 5969 uniaxial press (max. load 50 kN). The narrow central portion of the sample is enclosed in a Severn Thermal Solution split furnace housing a 16-mm diameter sapphire window to allow observation of cracking. B) Photograph of a post-experiment, jointed sample in the assembly (the split furnace is visible on each side of the sample). C) Experimental products showing the fractures developed during columnar jointing experiments.

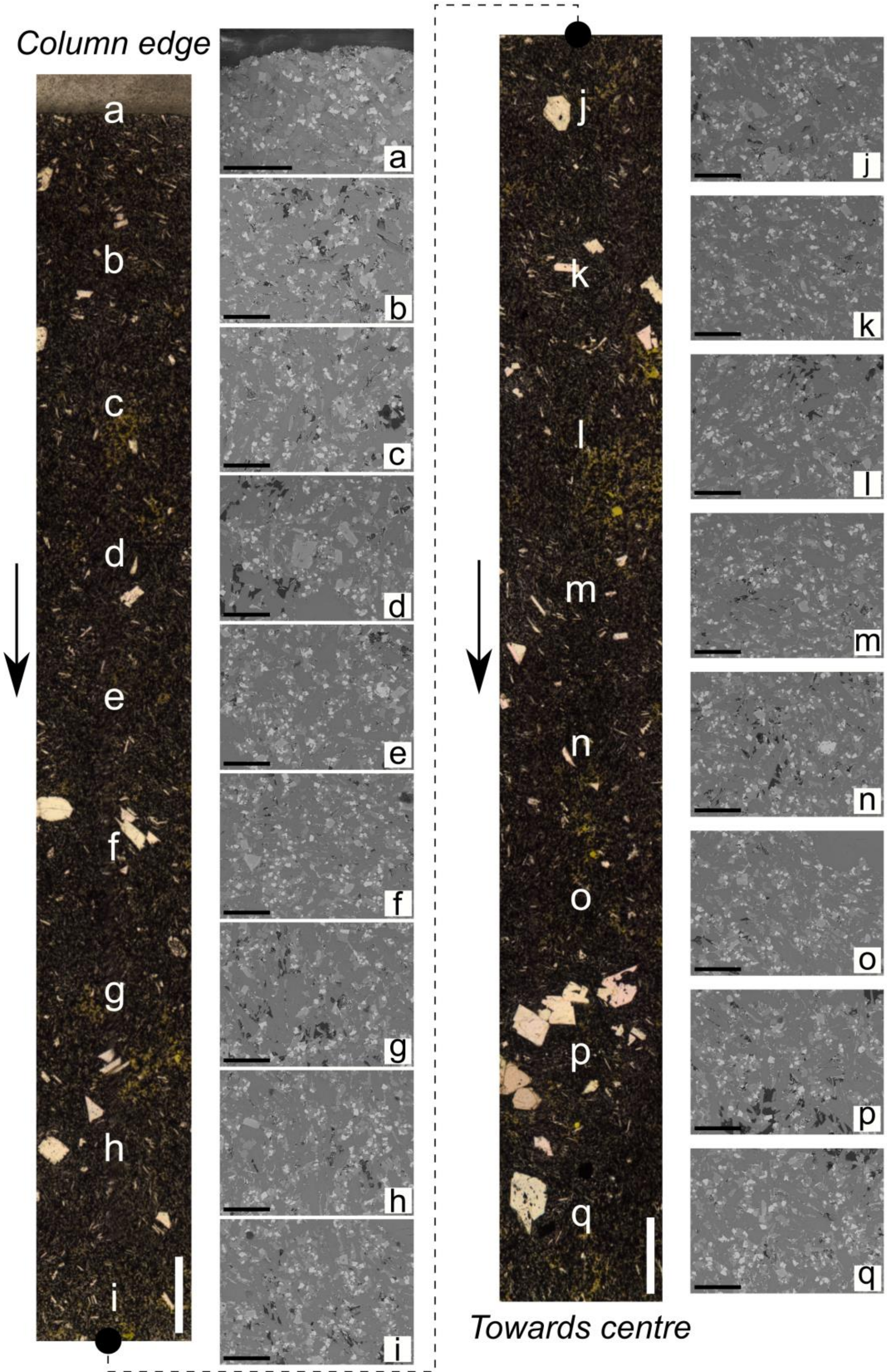

Supplementary Figure 2. Textural variation across a column in the Seljavellir basalt shown as a continuous section using optical photomicrographs, upon which labels correspond to the backscatter electron images (a to q) taken using a Hitachi TM3000 SEM using a 15 kV beam and 10 mm working distance. Although there is some variation in porosity distribution, the images show no systematic microstructural or textural changes across the column width, with the basalt made up of a homogeneous, fully crystalline groundmass (see supplementary Fig. 3), showing no evidence of a chilled margin or interstitial glass. White scale bars correspond to 5 mm in the optical images, whereas the black scale bars correspond to 200 µm on the SEM images.

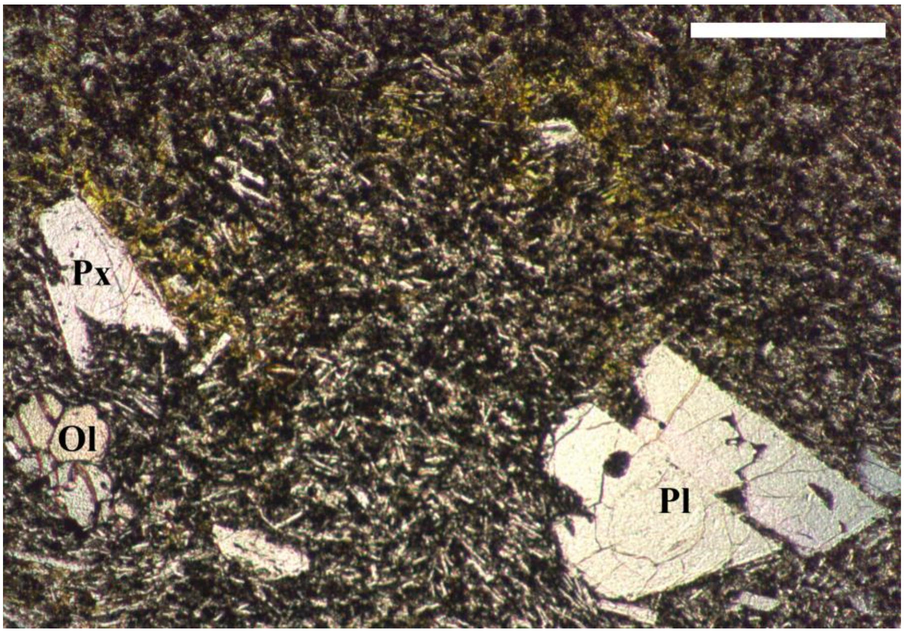

Supplementary Figure 3. Photomicrograph of the basalt from Seljavellir (Iceland), showing Olivine (Ol), Plagioclase (Pl) and Pyroxene (Px) crystals in a fine-grained groundmass containing plagioclase (light colour), pyroxene (dark grey) and iron oxides (black).

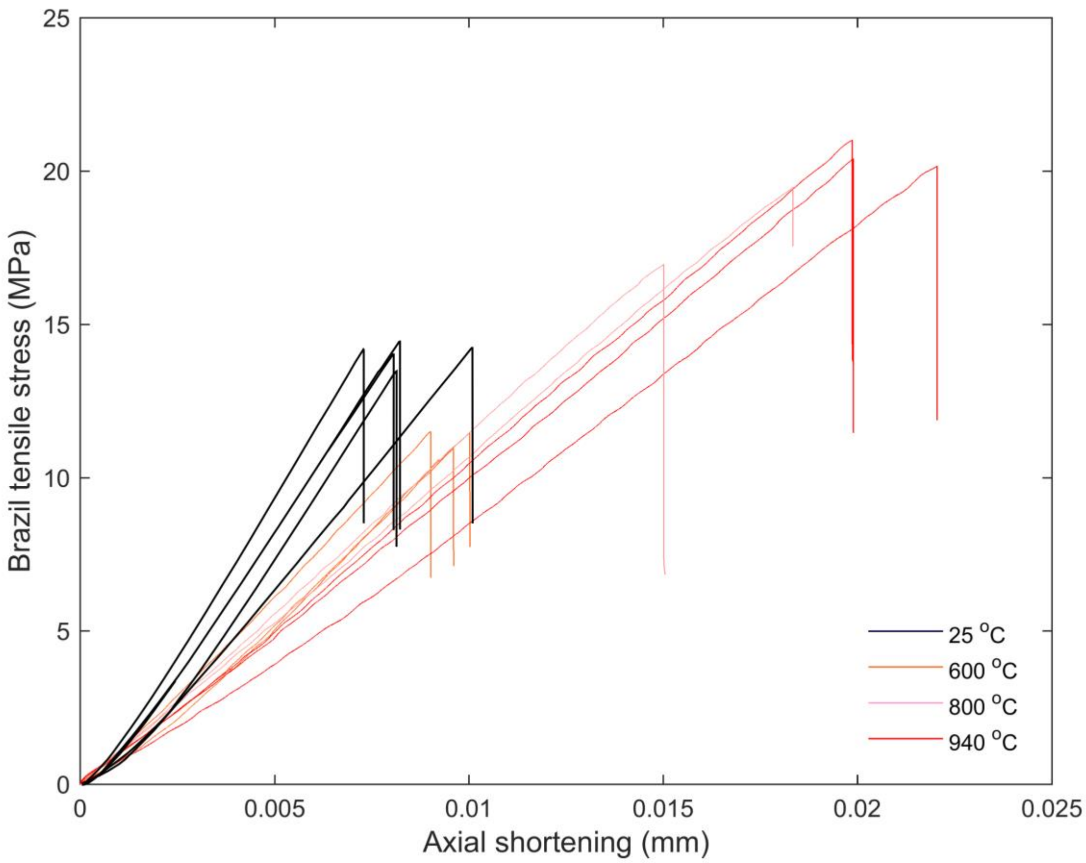

Supplementary Figure 4. Stress – axial shortening curves for Brazilian disc samples (40 mm diameter, 20 mm thickness) deformed diametrically at a rate of 0.4 $mm.s^{-1}$ at room temperature (black), 600 ºC (orange), 800 ºC (pink) and 940 ºC (red). The peak tensile stresses achieved determine lower (12 MPa) and upper (20 MPa) strength bounds for Seljavellir basalts.

Supplementary Tables

Oxides	Abundance (wt.%)	Standard deviation
**SiO_2**	48.40	0.24
**Al_2O_3**	14.35	0.14
**Fe_2O_3**	14.60	0.15
**MgO**	4.58	0.05
**CaO**	9.12	0.09
**Na_2O**	3.81	0.04
**K_2O**	1.00	0.01
**TiO_2**	3.70	0.04
**MnO**	0.22	0.02
**P_2O_5**	0.59	0.06
**SO_3**	0.01	0.01
**LOI**	-0.69	0.001

Supplementary Table 1. Bulk rock chemistry as obtained by x-ray fluorescence, performed at the University of Leicester (United Kingdom). LOI stands for loss on ignition and shows a minor weight gain associated with oxidation.
